# Integrating Social Determinants of Health With Treatment and Prevention: A New Tool to Assess Local Area Deprivation

**DOI:** 10.5888/pcd13.160221

**Published:** 2016-09-15

**Authors:** Andrew R. Maroko, Thao M. Doan, Peter S. Arno, Megan Hubel, Shirley Yi, Deborah Viola

**Affiliations:** Author Affiliations: Thao M. Doan, Deborah Viola, Center for Regional Healthcare Innovation, Westchester Medical Center Health Network, Hawthorne, New York; Peter S. Arno, Political Economy Research Institute, University of Massachusetts, Amherst, Massachusetts; Megan Hubel, Shirley Yi, CUNY School of Public Health and Health Policy, New York, New York.

## Abstract

We assessed the appropriate geographic scale to apply an area deprivation index (ADI), which reflects a geographic area’s level of socioeconomic deprivation and is associated with health outcomes, to identify and screen patients for social determinants of health. We estimated the relative strength of the association between the ADI at various geographic levels and a range of hospitalization rates by using age-adjusted odds ratios in an 8-county region of New York State. The 10-km local ADI estimates had the strongest associations with all hospitalization rates (higher odds ratios) followed by estimates at 20 km, 30 km, and the regional scale. A locally sensitive ADI is an ideal measure to identify and screen for the health care and social services needs and to advance the integration of social determinants of health with clinical treatment and disease prevention.

## Objectives

As the health care system shifts toward value-based planning and purchasing, new tools are needed to integrate social determinants of health into clinical and preventive care to improve population health and reduce health care disparities. An area deprivation index (ADI) is a multidimensional evaluation of a region’s socioeconomic conditions, which have been linked to health outcomes (1–6). We have refined the ADI, testing its association with health outcomes at various geographic levels. A simple-to-use tool such as the ADI can provide an efficient mechanism to alert health care providers to screen and refer patients for problems related to social determinants of health.

## Methods

We examined the relative strength of the association between a locally calibrated ADI with regional scale metrics and 4 hospitalization rates in New York’s Hudson Valley, an area that comprises 8 counties and is home to 2.3 million residents. The ADI, which is a composite measure of 17 census variables designed to describe socioeconomic disadvantage based on income, education, household characteristics, and housing (Box), was refined and provided by the University of Wisconsin’s Health Innovation Program and is available for the entire United States (7).

**Box. Census Variables in the Area Deprivation Index**

**Table:** 

Domain	Variable
Education	% Population aged 25 years or older with less than 9 years of education
	% Population aged 25 years or older with at least a high school diploma
	% Employed population aged 16 years or older in white-collar occupations

Income/employment	Median family income in US dollars
	Income disparity
	% Families below federal poverty level
	% Population below 150% of federal poverty level
	% Civilian labor force population aged 16 years and older who are unemployed

Housing	Median home value in US dollars
	Median gross rent in US dollars
	Median monthly mortgage in US dollars
	% Owner-occupied housing units
	% Occupied housing units without complete plumbing

Household characteristics	% Single-parent households with children younger than 18
	% Households without a motor vehicle
	% Households without a telephone
	% Households with more than 1 person per room

Hospitalizations for individual patients from 1999 through 2001 from the New York State Department of Health Statewide Planning and Research Cooperative System were averaged to match the ADI data for the year 2000. Major diagnostic categories were total hospital admissions, respiratory system (*International Classification of Diseases, 9th edition* [ICD-9], codes 460–519), circulatory system (ICD-9 codes 390–459), and mental disorders (ICD-9 codes 290–319). These data were available to us only in zip code aggregates, which were then converted to zip code tabulation areas (ZCTAs) to match our other data sets for analysis. Indirect age-adjustment was applied by using New York State as the standard population. We used ArcGIS 10.2 (ESRI) to spatialize and map ADI and health data.

**Figure 1:**
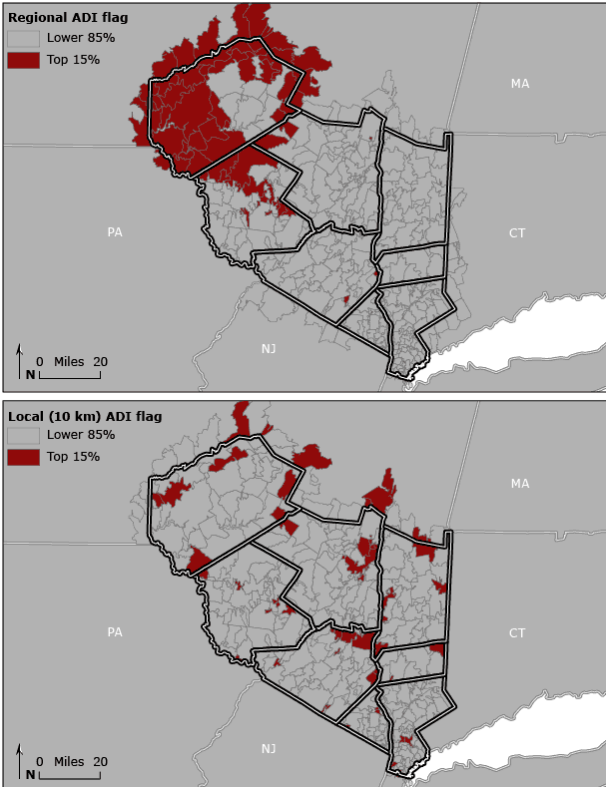
Regional versus local variation in the area deprivation index (ADI) in the Hudson Valley of New York. Dichotomized ADI values were calibrated on the basis of regional and local 10-km scales by zip code tabulation area.

ADI values were dichotomized as the top 15% (the highest level of deprivation) and the bottom 85% according to the national threshold of 15% established by Kind et al, who found this level to be closely associated with Medicare rehospitalization rates across the country (5). Regional-level ADI was characterized by recalculating percentiles and the 15% threshold by using data from the Hudson Valley study area. Local-scale ADI was calculated by assigning each ZCTA a percentile value for ADI relative to the ZCTAs within a 10-km, 20-km, and 30-km radius. This created a type of “moving window” across the study area. If the central ZCTA was in the top 15% of ADI values when compared with its neighbors, it was flagged as having a high level of local deprivation (Figure 1).

To validate ADI metrics, age-adjusted odds ratios were calculated by using SPSS (IBM Corp) on the various ADI measures serving as the exposures and hospitalizations for the selected major diagnostic categories serving as outcomes to assess the relative strengths of the associations.

## Results

**Figure 2:**
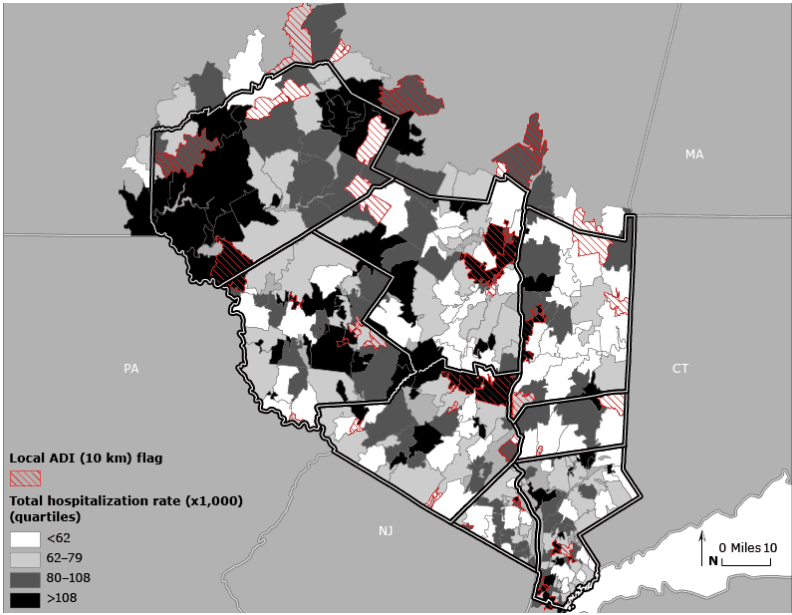
Local area deprivation index (ADI) values versus total hospitalization rate, Hudson Valley, New York. Zip code tabulation areas are indicated with cross hatching to depict local 10-km ADI values above the 15% threshold. Total hospitalization rates (1999–2000) are shown in quartiles. Maps of other health outcomes are available from the author upon request.

The 10-km local ADI estimates had the strongest associations with all hospitalization rates (higher odds ratios) followed by estimates at 20 km, 30 km, and the regional scale, respectively (Table). A locally adjusted ADI, at least for the Hudson Valley, was more strongly associated with health outcomes than those scaled at higher geographic levels. Figure 2 depicts hospitalization rates and local ADI values above the 15% threshold. 

**Table:** Associations Between Hospitalization Rates and the Area Deprivation Index^a^ (ADI), by Geographic Scale, in the 8-County^b^ Area of the Hudson Valley, New York State, 1999–2001^c^

ADI Measure^d^	Total Hospitalizations	Respiratory Hospitalizations	Circulatory Hospitalizations	Mental Health Hospitalizations
Local (10 km)	1.51 (1.50–1.53)	1.68 (1.64–1.73)	1.47 (1.44–1.51)	2.20 (2.14–2.26)
Local (20 km)	1.42 (1.41–1.44)	1.60 (1.56–1.65)	1.41 (1.37–1.44)	1.75 (1.70–1.80)
Local (30 km)	1.28 (1.27–1.29)	1.38 (1.34–1.42)	1.27 (1.23–1.30)	1.66 (1.62–1.71)
Regional	1.11 (1.09–1.14)	1.30 (1.22–1.38)	1.18 (1.11–1.25)	0.85 (0.79–0.92)

a The ADI is a composite measure of 17 census variables designed to describe socioeconomic disadvantage based on income, education, household characteristics, and housing.

b The 8 counties were Delaware, Dutchess, Orange, Rockland, Putnam, Sullivan, Ulster, and Westchester.

c All values are odds ratios (95% confidence intervals). The higher the odds ratio, the stronger the association between ADI measure and hospitalization rate. Confidence intervals may overestimate significance because of the large study area population.

d Local-scale ADI was calculated by assigning each zip code tabulation area (ZCTA) a percentile value for ADI relative to the ZCTAs within a 10-km, 20-km, and 30-km radius. If the central ZCTA was in the top 15% of ADI values when compared with its neighbors, it was flagged as having a high local level of deprivation.

## Discussion

These findings support the need for locally sensitive relative deprivation measures; national and regional approaches do not appear to properly model the potential impact of area deprivation on health outcomes in a smaller study area. These results are striking, but they should be updated and tested for their applicability to other parts of the United States. Using smaller, more homogeneous units of aggregation (eg, census tract) may improve these findings. Also, because this was an ecological study, it was not possible to disentangle the contextual and compositional factors that may be driving these associations. However, making a simple and reliable indicator of area deprivation available to health care providers could aid in providing useful and appropriate treatment. If aligned with a patient’s ZCTA, an indicator such as the ADI can facilitate referrals to local community resources and improve access to health and social services, particularly for underserved populations. Kind et al. suggested that “clinicians and health systems could, at the point of first contact, use the ADI to screen for patients returning to the most challenging environments. This would support the early targeting of more intensive transitional care services, prompt discussion of socioeconomic environment and need, and activate additional community resources for these patients” (5). Employing a dichotomized and automated “flag” for deprivation, particularly if incorporated into electronic health records, could ease the identification of social determinants of health issues and facilitate access to resources in a patient’s local community.

As large health care systems, including accountable care organizations, transition toward value-based planning and purchasing — in a shift from volume to value — identifying and addressing the social determinants of health of the populations they serve will become essential. In New York State, for example, beginning in 2018 Medicaid providers and their community-based partners will be required to implement, and will be reimbursed for, one or more interventions related to social determinants of health (8). Similar plans are underway in other states and nationally through the Medicare program (9,10). These types of initiatives, which will depend on reliable data such as a locally sensitive ADI, can improve access to health and social services in underserved populations, allow safety-net providers and other providers to more efficiently target their resources, and advance the integration of social determinants of health into clinical treatment and disease prevention.
